# The impact of ERBB-family germline single nucleotide polymorphisms on survival response to adjuvant trastuzumab treatment in HER2-positive breast cancer

**DOI:** 10.18632/oncotarget.12782

**Published:** 2016-10-20

**Authors:** Sinead Toomey, Stephen F. Madden, Simon J. Furney, Yue Fan, Mark McCormack, Carragh Stapleton, Mattia Cremona, Gianpiero L. Cavalleri, Malgorzata Milewska, Naomi Elster, Aoife Carr, Joanna Fay, Elaine W. Kay, Susan Kennedy, John Crown, William M. Gallagher, Bryan T. Hennessy, Alex J. Eustace

**Affiliations:** ^1^ Medical Oncology Group, Department of Molecular Medicine, Royal College of Surgeons in Ireland, Ireland; ^2^ Population Health Sciences Division, Royal College of Surgeons in Ireland, Ireland; ^3^ School of Medicine, University College Dublin, Ireland; ^4^ University College Dublin, UCD School of Biomolecular and Biomedical Science, Conway Institute of Biomolecular and Biomedical Research, Ireland; ^5^ Molecular and Cellular Therapeutics, Royal College of Surgeons in Ireland, Ireland; ^6^ Department of Histopathology, Beaumont Hospital, Dublin, Ireland; ^7^ St Vincent's University Hospital, Dublin, Ireland; ^8^ Department of Oncology, Beaumont Hospital, Dublin, Ireland

**Keywords:** ERBB-family germline mutations, single nucleotide polymorphisms, high depth sequencing, survival impact

## Abstract

**Background:**

Trastuzumab treatment for women with HER2-positive breast cancer (BC) resulted in the significant improvement of both relapse free survival (RFS) and overall survival (OS). However, many women who are classified as HER2-positive do not respond. Many studies have focused on the role of somatic mutations rather than germline polymorphisms in trastuzumab resistance.

**Results:**

We completed an Agena MassArray screen of 10 ERBB-family single nucleotide polymorphisms (SNPs) in 194 adjuvant trastuzumab treated HER2-positive BC patients. SNPs in EGFR genes have a significant association with RFS and OS. Patients with the minor allele of EGFR N158N had significantly worse OS (hazard ratio (HR) = 4.01, (confidence interval (CI) = 1.53– 10.69), *p* = 0.05) relative to those with either the heterozygous or wild-type (WT) allele. Patients with the minor allele of EGFR T903T (HR = 3.52, (CI = 1.38– 8.97), *p* = 0.05) had worse RFS relative to those with either the heterozygous or WT allele.

**Patients and methods:**

Using next generation sequencing (NGS) we identified ERBB-family (EGFR, HER2, HER3 and HER4) single nucleotide polymorphisms (SNPs) that occurred in 2 or more patients of a 32 HER2-positive BC patient cohort. Agena MassArray analysis confirmed the frequency of these SNPs in 194 women with HER2-positive BC who received trastuzumab in the adjuvant setting. Using Kaplan-Meier estimates and Cox regression analysis we correlated the presence of ERBB-family SNPs with both RFS and OS.

**Conclusions:**

The presence of germline ERBB-family SNPs may play an important role in how a patient responds to adjuvant trastuzumab, and clinical assessment of these SNPs by targeted genetic screening of patients' blood may be important to stratify patients for treatment.

## INTRODUCTION

Breast cancer (BC) remains the most common form of malignancy in women with over 25% of all cancers being diagnosed as BC in 2012 [1]. In HER2-positive BC, which accounts for approximately 20% of all human BCs, HER2 gene amplification and overexpression is associated with an aggressive phenotype and poor prognosis [2]. Trastuzumab, a monoclonal antibody targeted to HER2, has well established efficacy in the treatment of HER2-positive BC [3, 4]. However, a significant proportion of patients with the disease have tumors that initially do not respond or that acquire resistance to trastuzumab after an initial period of response [3, 4]. Many potential mechanisms of trastuzumab resistance in HER2-positive BC have been proposed which have been discussed in detail by us and others [2, 5]; including altered intracellular signaling involving loss of PTEN, reduced p27kip1, increased PI3K/Akt activity (e.g. PIK3CA mutations) or altered signaling via non-HER family receptor tyrosine kinases such as IGF1R.

However, few studies have been conducted to understand the role of innate resistance to trastuzumab. The advent of next generation sequencing (NGS) has allowed researchers access to the information stored in the genome. Whilst much focus has been targeted towards the somatic mutations in cancer, less attention has been focused on the role of germline single nucleotide polymorphisms (SNPs) and their role in cancer development and therapy response. In fact, recent studies have identified that SNPs can be biomarkers of the likelihood of developing cancer and several have been implicated in targeted therapy response and resistance [6].

ERBB-family genes which encode the HER family of proteins EGFR, HER2, HER3 and HER4 are commonly studied in HER2-positive BC, and some studies have identified the role of HER2 SNPs in response to trastuzumab [7]. Here we have determined the frequency of germline ERBB-family SNPs in HER-2 positive BC patients by NGS and correlated their genotype with the progression of HER2-positive BC and trastuzumab response.

## RESULTS

### Identification of ERBB-family SNPs in 194 HER2-positive BC patients

We identified 10 individual common ERBB-family SNPs present in at least 2 samples in our 194 HER2-positive BC patients (Table 1). 5 of the EGFR SNPs occurred in the kinase, transmembrane or growth factor receptor (GFR) domains of the protein. The EGFR-D994D SNP occurred in a non-defined domain of the protein. HER2-P1170A occurred in a non-defined domain of the protein, whilst HER2-I655V occurred in the transmembrane domain of the protein. The HER3 SNP R1116R occurred in the non-defined region of the protein, whilst HER3-I390I is found in the receptor ligand domain of the protein.

**Table 1:** Impact of ERBB-family SNPs on relapse free and overall survival of HER2-positive BC patients who have received adjuvant trastuzumab as part of their therapeutic regimen (*n* = 194)

Gene	Protein Domain	Accession number	SNP	R/MA allele	RFS HR (95% CI)	*P*-value	Adjusted *p*-value	OS HR (95% CI)	*P*-value	Adjusted *p*-value
**EGFR**	Receptor ligand	rs2072454	N158N	C/T	1.03 (0.43–2.48)	0.96	0.96	4.01 (1.53–10.69)	4.8 × 10^−3^	0.05
	GFR	rs2227983	T584T	T/A	0.90 (0.38–2.14)	0.80	0.89	1.51 (0.43–5.38)	0.52	0.95
	Trans membrane	rs10258429	H656H	C/T	0.72 (0.38–1.38)	0.32	0.78	1.41 (0.58–3.44)	0.44	0.95
	Protein kinase	rs1050171	Q787Q	G/A	1.17 (0.39–3.55)	0.78	0.89	1.11 (0.31–3.93)	0.88	0.95
	Protein kinase	rs1140475	T903T	C/T	**3.52 (1.38–8.97)**	**4.94** × **10^−3^**	**0.05**	1.05 (0.25–4.40)	0.95	0.95
	NDD	rs2293347	D994D	G/C	1.38 (0.66–2.91)	0.39	0.78	**2.60 (1.16–5.82)**	**0.02**	**0.08**
**ERBB2**	Trans membrane	rs1136201	I655V	A/G	**1.79 (1.00–3.19)**	**0.05**	**0.17**	1.07 (0.52–2.23)	0.85	0.95
	NDD	rs1058808	P1170A	C/G	0.68 (0.23–1.95)	0.47	0.78	0.87 (0.29–2.57)	0.80	0.95
**ERBB3**	Receptor ligand	rs2229046	I390I	T/C	**1.99 (1.01–3.90)**	**0.04**	**0.17**	1.85 (0.81–3.67)	0.15	0.51
	NDD	rs2271189	R1116R	G/A	1.28 (0.54–3.07)	0.57	0.81	0.94 (0.32–2.70)	0.90	0.95

SNP = Single nucleotide polymorphism; RFS = Relapse Free Survival; OS = overall survival; HR = Hazard Ratio; R = reference allele; MA = minor allele.

### Association between ERBB-family polymorphisms and survival in HER2-positive patients who received trastuzumab as part of their therapeutic regimen

**Figure 1:**
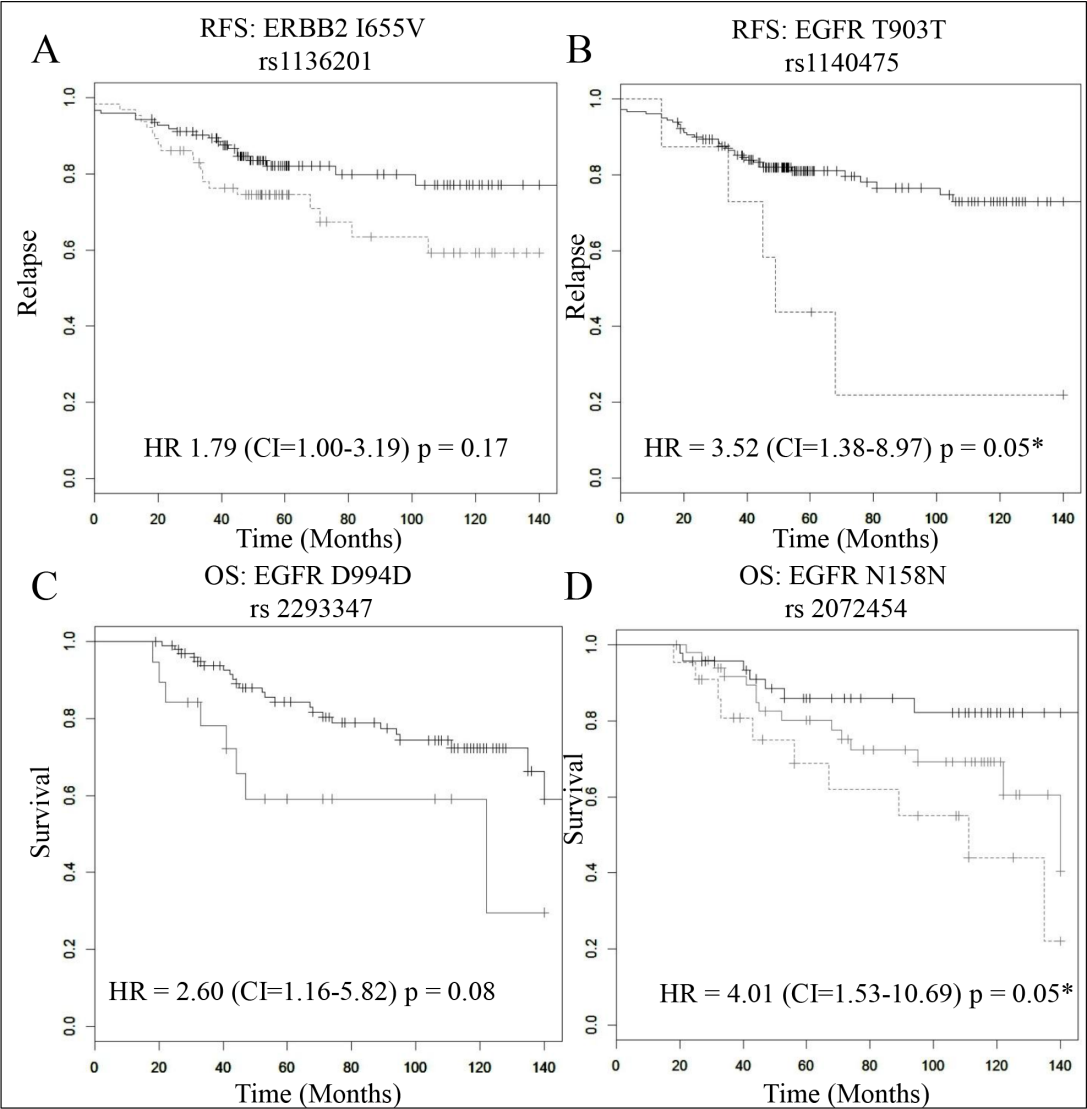
The prognostic role of 4 SNPs in trastuzumab treated primary tumours (*n* = 194) In each plot the black, grey and dotted grey lines represent the samples with the wild type (reference), heterozygous and mutant (minor) alleles respectively. Kaplan Meier estimates of (**A**) the ERBB2-I655V SNP where RFS is the survival endpoint (HR = 1.79 (CI = 1.00–3.19), *p* = 0.17), (**B**) the EGFR-T903T SNP, where RFS is the survival endpoint (HR = 3.52, (CI = 1.38–8.97), *p* = 0.05), (**C**) the EGFR-D994D SNP, where OS is the survival endpoint (HR = 3.13 (CI = 1.19–8.33), *p* = 0.08), and in this case there were no samples with the minor allele (no green line) and (**D**) the EGFR-N151N SNP, where OS is the survival endpoint (HR = 4.01 (CI = 1.53–10.69), *p* = 0.05). * indicates a significant *p-value* after multiple testing correction.

**Figure 2:**
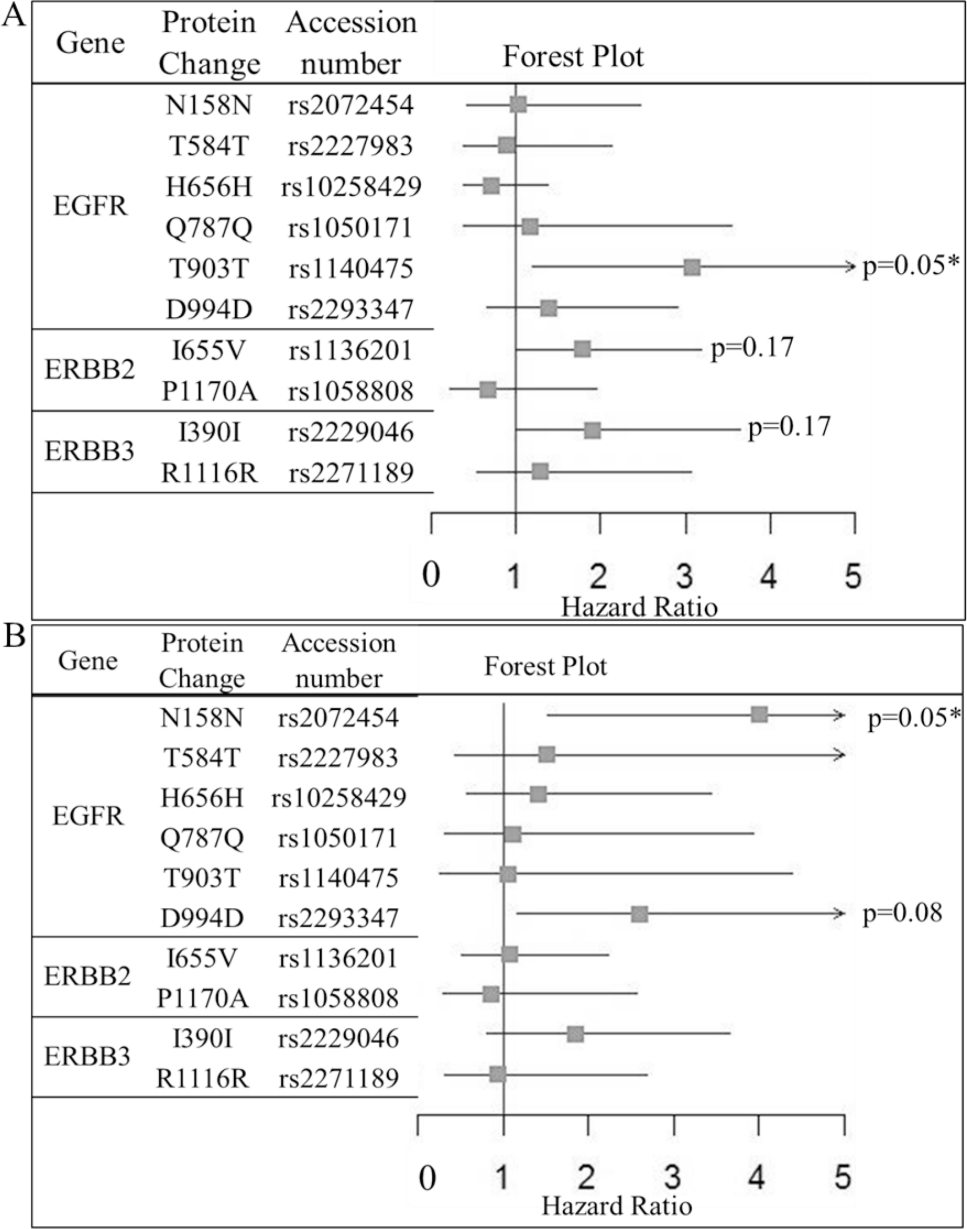
Forest plot of the impact of ERBB-family SNPs on (A) relapse free survival and (B) Overall survival of HER2-positive BC patients who have received adjuvant trastuzumab as part of their therapeutic regimen (*n* = 194) Hazard ratios, confidence intervals and *p-value*s were calculated using Cox regression analysis, where an adjusted *p-value* of < = 0.05 was considered significant. Significant SNPs are marked on the plot.

Patients with the HER2 SNP I655V who were homozygous for the minor allele (G) were significantly more likely to have a worse RFS rate than those who were homozygous for the WT allele (A) or were heterozygous for the allele (A/G) (HR = 1.79 (CI = 1.00–3.19), *p* = 0.05, Table 1, Figure 1(A)). However, when adjusting for multiple testing HER2-I655V did not remain significant (*p* = 0.17) (Table 1, Figure 2(A)). After multivariate analysis the difference in the rate of RFS remains significant when adjusted for ER status, Age and LN status (HR = 2.36 (CI = 1.02–5.50), *p* = 0.04). Again however, no benefit was observed for OS for either of the alleles tested. This is consistent with previous findings by Han *et al.*, 2014 [8]. We analyzed the frequency differences of the ERBB-family SNPs between the Irish, UK and Chinese populations to determine if ERBB SNPs differ greatly between the two populations (Table 2). We found that the ERBB-I655V SNP did not differ significantly between the Chinese Han, Irish or UK populations, providing added support to the hypothesis that the minor allele of ERBB2-I655V has a negative impact on response to adjuvant trastuzumab therapy.

**Table 2:** Comparison of the allele frequencies of ERBB-family SNPs between the Irish HER2-Positive BC cohort and the 1000 Genomes UK and Chinese Han cohorts

Gene	Protein Change	Accession number	HER2-IRE MAF *n* = 478	1kG-GBR MAF *n* = 182	1kG-CHB MAF *n* = 206	HER2 IRE vs 1kG GBR		HER2 IRE vs 1kG CHB	
						*P*-value	Adjusted *P*-value	*P*-value	Adjusted *P*-value
**EGFR**	**N158N**	rs2072454	0.416	0.54	0.44	0.014	0.04	0.566	0.57
	**T584T**	rs2227984	0.125	0.3	0.48	9.12x^e-06^	4.56x^e-05^	1.27x^e-16^	1.91x^e-15^
	**H656H**	rs10258429	0.088	0.07	0.05	0.591	0.81	0.135	0.17
	**Q787Q**	rs1050171	0.357	0.64	0.17	9.80x^e-09^	7.35x^e-08^	9.86x^e-06^	2.11x^e-05^
	**T903T**	rs1140475	0.125	0.11	0.05	0.760	0.88	0.007	0.01
	**D994D**	rs2293347	0.092	0.07	0.26	0.481	0.72	2.18x^e-06^	5.45x^e-06^
**ERBB2**	**I655V**	rs1136201	0.247	0.27	0.17	0.653	0.82	0.049	0.07
	**P1170A**	rs1058808	0.275	0.65	0.52	2.34x^e-14^	3.50x^e-13^	2.02x^e-07^	1.01x^e-06^
**ERBB3**	**I390I**	rs2229046	0.09	0.04	0	0.034	0.08	1.40x^e-06^	4.21x^e-06^
	**R1116R**	rs2271189	0.42	0.42	0.38	0.921	0.99	0.438	0.47

MAF = Mutant Allele Frequency; IRE = Ireland (*n* = 239); 1 kG GBR = 1000 Genomes Study participants from Great Britain (*n* = 91); 1kG CHB = 1000 Genomes Study participants from Beijing, China (*n* = 103).

We also found that patients who received adjuvant trastuzumab and were heterozygous for the allele (T/C) were significantly more likely to have a worse RFS relative to those that were homozygous for the reference allele (T) (HR = 1.99 (CI = 1.01–3.90), *p* = 0.04) (Table 1). However, after multivariate analysis or correction for multiple testing (*p* = 0.17) ERBB3-I390I did not remain significant.

Patients who received adjuvant trastuzumab and were homozygous for the minor allele (T) of the EGFR SNP T903T were found to have worse RFS relative to those who were homozygous for the reference allele (C) or were heterozygous for the allele (C/T) (HR = 3.52, (CI = 1.38–8.97), *p* = 4.94 × 10^−3^, Table 1, Figure 1(B)). After correction for multiple testing T903T remained significant (*p* = 0.05) (Table 1, Figure 2 (A)). Multivariate analysis of the impact of adjuvant trastuzumab on RFS in the EGFR SNP T903T was still significant when adjusted for ER status, tumor grade and Age (HR = 6.51 (CI = 1.98– 21.36), *p* = 0.01). The impact of EGFR-T903T on RFS survival did not extend to a significant benefit in OS.

We also found in our population two EGFR SNPs (D994D and N158N) that were associated with better OS when patients were treated with adjuvant trastuzumab. Patients who were homozygous for the minor allele (C) of D994D were significantly more likely to have a worse OS rate than those who were homozygous for the reference allele (G) (HR = 2.60 (CI = 1.16–5.82), *p* = 0.02), Table 1, Figure 1(C)). After multivariate analysis however we found that EGFR D994D did not remain significant (*p* = 0.08). When we performed multivariate analysis we found that OS impact of the EGFR SNP D994D is still significant when adjusted for ER status, grade and age (HR = 3.46 (CI = 1.10–10.90), *p* = 0.02).

Patients who received trastuzumab and were homozygous for the reference allele (C) or had the heterozygous allele (C/T) in the EGFR SNP N158N were significantly more likely to have better OS than those who had the minor allele (T) (HR = 4.01 (CI = 1.53–10.69), *p* = 4.8 × 10^−3^, Table 1, Figure 1(D)). Multivariate analysis reveals that the OS impact of the EGFR SNP N158N is still significant when adjusted for ER status, age and tumor grade (HR = 3.47 (CI = 1.11–10.90), *p* = 0.03). After correcting for multiple testing EGFR- N158N remained significant (*p* = 0.05) (Table 1, Figure 2(B)). Neither EGFR-D994D nor N158N had an impact on RFS.

## DISCUSSION

Synonymous mutations have been well established as substantial contributors to human disease [9]. Whilst trastuzumab revolutionized the treatment of HER2-positive BC, many women either receive no initial benefit from the treatment or subsequently develop resistance. Many studies focus on the role of somatic mutations in acquired and innate resistance to trastuzumab [5] however few have analyzed the impact of germline SNPs. Eight of the 10 ERBB-family SNPs we identified are synonymous. Our analysis found that patients who had specific EGFR and HER2 family SNPs had worse RFS and OS.

Patients who had the non-synonymous HER2-I655V SNP and who were homozygous for the minor allele G were significantly more likely to have a worse RFS than those patients who had the major allele A, however the result did not survive multiple testing. This result is consistent with the Han *et al.* study of 2014 [8] which found that the minor allele of HER2-I655V resulted in worse RFS of Chinese HER2-positive BC patients. Our analysis found no significant difference in allele frequency of the ERBB2-I655V SNP between the Chinese Han and UK/Irish populations, providing further support that the minor allele of ERBB2-I655V may be a negative prognostic marker of adjuvant trastuzumab response in women who suffer from HER2-positive BC. Interestingly, neither Han *et al's.* [8] nor our study found that patients with either the minor or heterozygous allele had a worse OS relative to those that were WT allele. HER2-I655V has also been implicated in being causative of HER2-positive BC but the evidence is inconclusive [7, 10, 11]. However, recent studies which implicate the minor allele of HER2-I655V in trastuzumab cardiotoxicity have been more conclusive with the valine allele being associated with increased likelihood of cardiotoxicity [12, 13].

In addition, patients who received trastuzumab and had the minor allele (T) of EGFR-T903T were significantly more likely to have a worse RFS survival relative to those who were either heterozygous for the allele (C/T) or who had the major allele (C). However the impact of the SNP was not associated with a worse OS relative to patients who had both the heterozygous or major allele. Interestingly no previous study has implicated the EGFR-T903T SNP with any prognostic or predictive role, but previous studies have shown that synonymous SNPs such as this can impact a cell's ability to respond to therapeutics [14]. Indeed, previous studies have shown that synonymous mutations affect splicing accuracy, translation fidelity, mRNA structure and protein folding, all of which of the potential to contribute to drug response [14]. Supporting this hypothesis EGFR-D994D had been previously demonstrated to functionally impact the rate of protein translation [14].

The EGFR-T903T and HER2-I655V SNPs occur in the protein kinase and trans-membrane domains of the protein respectively. Alterations in these protein domains have been long associated with functional impact on cellular signaling [15, 16]. Future proteomic analysis of the impact of synonymous SNPs will provide important functional information as to how these SNPs impact on cellular signaling.

Previous studies of EGFR-D994D found that lung patients who received gefitinib as part of their therapeutic regimen were more likely to have a worse OS [17]. Separate studies have shown that EGFR-D994D plays a role in lung cancer susceptibility and that it also plays a role in sensitivity to chemotherapy [9, 18]. Our study whilst borderline significant showed that D994D had an impact on OS in trastuzumab treated HER2-positive BC patients. The published evidence of gefitinib sensitivity in lung cancer and our observation of trastuzumab sensitivity in HER2-positive BC, suggests that this SNP may be a general rather than a therapeutic specific marker.

Our results suggest that being either homozygous for the minor allele or being heterozygous for the allele (C/T) for the SNP EGFR-N158N is significantly associated with a worse OS relative to those that are homozygous for the reference allele. The SNP EGFR-N158N has previously been associated with a higher risk of developing head and neck cancer [6], but its role in the development of lung cancer is unclear. Our study is the first to associate EGFR-N158N with worse OS in women who have HER2-positive BC. The impact of EGFR and ERBB2 SNPs warrant further study in a larger cohort of HER2-positive BC patient samples, to ascertain the impact of common ERBB2 and EGFR SNPs on trastuzumab response and survival in HER2-positive BC.

In conclusion, we have determined via high depth NGS the most common SNPs which occur in the ERBB-family genes of women who have HER2-positive BC. We found that 2 different SNPs in the HER2 and EGFR genes play a negative role in both the response to trastuzumab and OS. Overall our results indicate that SNPs which occur in genes which are frequently amplified or mutated in HER2-positive cancer may play a role in how a patient responds to therapies to treat the disease. The use of targeted genetic screening of patient's blood may allow for the stratification of patients who will benefit from adjuvant trastuzumab treatment.

## MATERIALS AND METHODS

### Patients

A total of 122 patients with operable primary BC were treated at Beaumont Hospital and St. Vincent's University Hospital between 1996 and 2012. 72 patients from the TCHL study were also included in the analysis. TCHL (NCT01485926) was a Phase II neo-adjuvant study assessing TCH (docetaxel, carboplatin and trastuzumab) and TCHL (TCH and lapatinib) in early-stage HER-2 positive BC [19]. This study, approved by the Research Ethics Committees of all hospitals, included women who are confirmed as clinically HER2-positive by a 3+ HER2 immunohistochemistry score and or/ a FISH ratio of > 2. Detailed clinical information is available in Supplementary Tables 1 of the 194 patients who received trastuzumab in the adjuvant setting and were included in the survival analysis.

### High depth sequencing

In our initial screen for ERBB-family related SNPs, we selected for high depth sequencing 32 tumor formalin-fixed, paraffin-embedded (FFPE) samples from patients with HER2-positive BC. Haematoxylin and eosin sections cut from the patient's FFPE surgical blocks were analyzed by a pathologist for tumor content and those with > 50% tumor cellularity had a further 7*10 μM sections cut, from which DNA was extracted using the Qiagen QIAamp DNA FFPE kit. Beta-globin PCR was carried out on all samples to ensure that DNA fragment lengths were greater than 300 bp. Samples were prepped and sequenced by Source Biosciences using an Agilent SureSelect panel using SureSelect QXT chemistry. The custom library designed comprised of the ERBB-family genes and included 132 regions. The total library size was 50 kbp, with average coverage of 98.87%. The custom library included coding exons of the target genes. The constructed libraries were sequenced using an Illumina MiSeq Sequencer.

### Sequencing analysis

Reads were trimmed using Trimmomatic [20] and aligned with BWA mem (version 0.7.5a-r405: http://bio-bwa.sourceforge.net/) under default parameters. Duplicate reads were marked by Picard tools (http://broadinstitute.github.io/picard/) and local realignment and base recalibration were conducted with GATK [21] (version v3.2–2-gec30cee, human genome version 19). Pileup files were generated using Samtools [22] (version 0.1.19–44428cd), excluding reads with mapping quality < 20, and variants were called with Varscan [23] (version v2.3.7) at positions with coverage ≥ 20. Variants were annotated by Variant Effect Predictor [24].

### Agena massarray

Mass spectrometry-based genotyping (Agena MassARRAY, Agena Bioscience, San Diego, CA) was applied to confirm the allele calls of the 10 SNPs which are listed in Table 1 from the NGS screen of ERBB-family genes. We also then tested a further 162 HER2-positive BC patient samples to increase our test population to 194 patients focusing only on the 10 most significant SNPs from the initial 32 sample screen. Reactions where > 15% of the resultant mass ran in the mutant site were scored as positive.

### Statistical analysis

Survival analysis was only performed using data from trastuzumab treated samples, using both relapse free survival (RFS) and overall survival (OS) as the survival endpoints (*n* = 194). Survival curves are based on Kaplan-Meier estimates and the log-rank *p-value* is shown for difference in survival. Cox regression analysis was used to calculate hazard ratios and perform multivariate analysis. The R package survival is used to calculate and plot the Kaplan-Meier survival curves. A fisher exact test was used to compare SNP frequencies between the different public datasets. All *p-value*s are adjusted for multiple testing using the Benjamini-Hochberg method [25]. All calculations are carried out in the R statistical environment (http://cran.r-project.org/).

## SUPPLEMENTARY MATERIALS FIGURES



## References

[1] GLOBOCAN Breast Cancer estimated Incidence, mortality and prevalence Worldwide in 2012.

[2] Hennessy BT, Smith DL, Ram PT, Lu Y, Mills GB (2005). Exploiting the PI3K/AKT pathway for cancer drug discovery. Nat Rev Drug Discov.

[3] Slamon DJ, Leyland-Jones B, Shak S, Fuchs H, Paton V, Bajamonde A, Fleming T, Eiermann W, Wolter J, Pegram M, Baselga J, Norton L (2001). Use of chemotherapy plus a monoclonal antibody against HER2 for metastatic breast cancer that overexpresses HER2. N Engl J Med.

[4] Piccart-Gebhart MJ, Procter M, Leyland-Jones B, Goldhirsch A, Untch M, Smith I, Gianni L, Baselga J, Bell R, Jackisch C, Cameron D, Dowsett M, Barrios CH (2005). Trastuzumab after adjuvant chemotherapy in HER2-positive breast cancer. N Engl J Med.

[5] Elster N, Collins DM, Toomey S, Crown J, Eustace AJ, Hennessy BT (2015). HER2-family signalling mechanisms, clinical implications and targeting in breast cancer. Breast Cancer Res Treat.

[6] Fung C, Zhou P, Joyce S, Trent K, Yuan JM, Grandis JR, Weissfeld JL, Romkes M, Weeks DE, Egloff AM (2015). Identification of epidermal growth factor receptor (EGFR) genetic variants that modify risk for head and neck squamous cell carcinoma. Cancer Lett.

[7] Alaoui-Jamali MA, Morand GB, da Silva SD (2015). ErbB polymorphisms: insights and implications for response to targeted cancer therapeutics. Front Genet.

[8] Han X, Diao L, Xu Y, Xue W, Ouyang T, Li J, Wang T, Fan Z, Fan T, Lin B, Xie Y (2014). Association between the HER2 Ile655Val polymorphism and response to trastuzumab in women with operable primary breast cancer. Ann Oncol.

[9] Choi JE, Park SH, Kim KM, Lee WK, Kam S, Cha SI, Kim CH, Kang YM, Kim YC, Han SB, Jung TH, Park JY (2007). Polymorphisms in the epidermal growth factor receptor gene and the risk of primary lung cancer: a case-control study. BMC Cancer.

[10] Dahabreh IJ, Murray S (2011). Lack of replication for the association between HER2 I655V polymorphism and breast cancer risk: a systematic review and meta-analysis. Cancer Epidemiol.

[11] Breyer JP, Sanders ME, Airey DC, Cai Q, Yaspan BL, Schuyler PA, Dai Q, Boulos F, Olivares MG, Bradley KM, Gao YT, Page DL, Dupont WD (2009). Heritable variation of ERBB2 and breast cancer risk. Cancer Epidemiol Biomarkers Prev.

[12] Roca L, Dieras V, Roche H, Lappartient E, Kerbrat P, Cany L, Chieze S, Canon JL, Spielmann M, Penault-Llorca F, Martin AL, Mesleard C, Lemonnier J (2013). Correlation of HER2, FCGR2A, and FCGR3A gene polymorphisms with trastuzumab related cardiac toxicity and efficacy in a subgroup of patients from UNICANCER-PACS 04 trial. Breast Cancer Res Treat.

[13] Beauclair S, Formento P, Fischel JL, Lescaut W, Largillier R, Chamorey E, Hofman P, Ferrero JM, Pages G, Milano G (2007). Role of the HER2 [Ile655Val] genetic polymorphism in tumorogenesis and in the risk of trastuzumab-related cardiotoxicity. Ann Oncol.

[14] Sauna ZE, Kimchi-Sarfaty C (2011). Understanding the contribution of synonymous mutations to human disease. Nat Rev Genet.

[15] Moasser MM (2007). The oncogene HER2: its signaling and transforming functions and its role in human cancer pathogenesis. Oncogene.

[16] Oda K, Matsuoka Y, Funahashi A, Kitano H (2005). A comprehensive pathway map of epidermal growth factor receptor signaling. Mol Syst Biol.

[17] Zhang L, Yuan X, Chen Y, Du XJ, Yu S, Yang M (2013). Role of EGFR SNPs in survival of advanced lung adenocarcinoma patients treated with Gefitinib. Gene.

[18] Takahashi H, Kaniwa N, Saito Y, Sai K, Hamaguchi T, Shirao K, Shimada Y, Matsumura Y, Ohtsu A, Yoshino T, Takahashi A, Odaka Y, Okuyama M (2013). Identification of a candidate single-nucleotide polymorphism related to chemotherapeutic response through a combination of knowledge-based algorithm and hypothesis-free genomic data. J Biosci Bioeng.

[19] Crown J, Coate L, Keane M, Kennedy J, O'Reilly S, Kelly C, O'Connor M, Martin M, Duffy K, Murphy C, Walshe J, O'Shea T, Moulton B (2013). Randomized phase II study of pre-operative docetaxel, carboplatin with trastuzumab (TCH) and/or/lapatinib (L) in HER-2 positive (H+) breast cancer patients (BC pts). ICORG 10-05. Cancer Res.

[20] Bolger AM, Lohse M, Usadel B (2014). Trimmomatic: a flexible trimmer for Illumina sequence data. Bioinformatics.

[21] DePristo MA, Banks E, Poplin R, Garimella KV, Maguire JR, Hartl C, Philippakis AA, del Angel G, Rivas MA, Hanna M, McKenna A, Fennell TJ, Kernytsky AM (2011). A framework for variation discovery and genotyping using next-generation DNA sequencing data. Nat Genet.

[22] Li H, Handsaker B, Wysoker A, Fennell T, Ruan J, Homer N, Marth G, Abecasis G, Durbin R, Genome Project Data Processing S (2009). The Sequence Alignment/Map format and SAMtools. Bioinformatics.

[23] Koboldt DC, Zhang Q, Larson DE, Shen D, McLellan MD, Lin L, Miller CA, Mardis ER, Ding L, Wilson RK (2012). VarScan 2: somatic mutation and copy number alteration discovery in cancer by exome sequencing. Genome Res.

[24] McLaren W, Pritchard B, Rios D, Chen Y, Flicek P, Cunningham F (2010). Deriving the consequences of genomic variants with the Ensembl API and SNP Effect Predictor. Bioinformatics.

[25] Benjamini Y, Hochberg Y (1995). Controlling the False Discovery Rate - a Practical and Powerful Approach to Multiple Testing. Journal of the Royal Statistical Society Series B-Methodological.

