# Supplementary material for: The impact of ERBB-family germline single nucleotide polymorphisms on survival response to adjuvant trastuzumab treatment in HER2-positive breast cancer
Source: Oncotarget. 2016 Oct 20;7(46):75518–25. doi: 10.18632/oncotarget.12782 (PMC5342757; doi:10.18632/oncotarget.12782)
Supplement: Supplementary file 1 [file oncotarget-07-75518-s001.pdf]

The impact of ERBB-family germline single nucleotide polymorphisms on survival response to adjuvant trastuzumab treatment in HER2-positive breast cancer

Supplementary Materials

Supplementary Table S1: Summary of patient characteristics of the 194 HER2-positive BC patients used in this study from the TCHL study (NCT01485926) and Beaumont and St Vincent's University Hospitals

Feature	Sample Number (%)
**Mean Age ± SD (years)**	51 ± 12
**Grade**	
I	4 (2)
II	40 (21)
III	66 (34)
Unknown	84 (43)
**LN Status**	
Positive	58 (30)
Negative	53 (27)
Unknown	83 (43)
**ER Status**	
Positive	118 (61)
Negative	72 (37)
Unknown	4 (2)
**PR Status**	
Positive	54 (28)
Negative	95 (49)
Unknown	45 (23)
**OS ± SD (months)**	81 ± 44
**PFS ± SD (months)**	64 ± 39

OS = Overall survival; PFS = Progression Free Survival; SD = Standard Deviation; LN = Lymph Node; ER = Oestrogen Receptor; PR = Progesterone Receptor.
